# Prevalence and duration of non‐motor symptoms in prodromal Parkinson's disease

**DOI:** 10.1111/ene.13919

**Published:** 2019-03-01

**Authors:** R. Durcan, L. Wiblin, R. A. Lawson, T. K. Khoo, A. J. Yarnall, G. W. Duncan, D. J. Brooks, N. Pavese, D. J. Burn, the ICICLE‐PD Study Group

**Affiliations:** ^1^ Institute of Neuroscience Newcastle University Newcastle Upon Tyne UK; ^2^ School of Medicine and Menzies Health Institute Queensland Griffith University Nathan QLD Australia; ^3^ School of Medicine University of Wollongong Wollongong NSW Australia; ^4^ Centre for Clinical Brain Sciences University of Edinburgh Edinburgh UK; ^5^ Department of Nuclear Medicine and PET Centre Aarhus University Hospital Aarhus Denmark; ^6^ Faculty of Medical Science Newcastle University Newcastle Upon Tyne UK

**Keywords:** gender, motor, non‐motor symptoms, Parkinson's, prodromal

## Abstract

**Background and purpose:**

The prevalence and duration of non‐motor symptoms (NMS) in prodromal Parkinson's disease (PD) has not been extensively studied. The aim of this study was to determine the prevalence and duration of prodromal NMS (pNMS) in a cohort of patients with recently diagnosed PD.

**Methods:**

We evaluated the prevalence and duration of pNMS in patients with early PD (*n* = 154). NMS were screened for using the Non‐Motor Symptom Questionnaire (NMSQuest). We subtracted the duration of the presence of each individual NMS reported from the duration of the earliest motor symptom. NMS whose duration preceded the duration of motor symptoms were considered a pNMS. Individual pNMS were then grouped into relevant pNMS clusters based on the NMSQuest domains. Motor subtypes were defined as tremor dominant, postural instability gait difficulty (PIGD) and indeterminate type according to the Movement Disorder Society Unified Parkinson's Disease Rating Scale revision.

**Results:**

Prodromal NMS were experienced by 90.3% of patients with PD and the median number experienced was 4 (interquartile range, 2–7). A gender difference existed in the pNMS experienced, with males reporting more sexual dysfunction, forgetfulness and dream re‐enactment, whereas females reported more unexplained weight change and anxiety. There was a significant association between any prodromal gastrointestinal symptoms [odds ratio (OR), 2.30; 95% confidence interval (CI), 1.08–4.89, *P* = 0.03] and urinary symptoms (OR, 2.54; 95% CI, 1.19–5.35, *P* = 0.016) and the PIGD phenotype. Further analysis revealed that total pNMS were not significantly associated with the PIGD phenotype (OR, 1.10; 95% CI, 0.99–1.21, *P* = 0.068).

**Conclusions:**

Prodromal NMS are common and a gender difference in pNMS experienced in prodromal PD may exist. The PIGD phenotype had a higher prevalence of prodromal gastrointestinal and urinary tract symptoms.

## Introduction

Clinically, Parkinson's disease (PD) has been defined by the presence of motor deficits such as bradykinesia, tremor, rigidity and postural instability 1. However, patients with PD also experience a variety of other non‐motor symptoms (NMS) such as mood disorders, autonomic disturbances, cognitive impairment and sleep dysfunction throughout their disease trajectory.

Non‐motor symptoms can precede motor symptoms, indicating that a prodromal symptomatic stage exists in PD. The presence of hyposmia, constipation, depression and idiopathic rapid eye movement sleep behaviour disorder are well‐established symptoms that significantly increase the risk of development of PD 2.

The emerging concept of a prodromal phase of PD has led the International Parkinson and Movement Disorder Society task force to propose a new definition of PD; this definition would not base the diagnosis solely on motor symptoms but would also incorporate NMS 3. However, there is a paucity of research investigating the frequency and time of onset of NMS before the onset of the motor phase of PD. Furthermore, little is known about the relationship between particular clusters of prodromal NMS (pNMS) and the development of subsequent PD motor phenotypes, i.e. tremor dominant (TD), postural instability gait difficulty (PIGD) or indeterminate type 4.

We aimed to explore the frequency and time of onset of NMS before motor symptoms (pNMS) and the role of gender on pNMS experienced. We hypothesized that distinct clusters of pNMS would be associated with PIGD motor phenotype.

## Methods

### Patient eligibility criteria and recruitment

Patients with recently diagnosed PD from Newcastle upon Tyne and Gateshead were invited to take part in the study between June 2009 and December 2011 as part of the Incidence of Cognitive Impairment in Cohorts with Longitudinal Evaluation‐Parkinson's Disease (ICICLE‐PD) study 5.

All participants were diagnosed by a movement disorders specialist according to the UK Brain Bank criteria 1. Exclusion criteria comprised the following: drug‐induced parkinsonism secondary to exposure to dopamine receptor blocking agent at the onset of symptoms; vascular parkinsonism; and atypical forms of parkinsonism such as progressive supranuclear palsy, multiple system atrophy or corticobasal degeneration, according to accepted diagnostic criteria 6. Participants were also excluded if they had insufficient working knowledge of English, defined as being unable to perform the assessments and questionnaires in the opinion of the assessor, or significant memory impairment or dementia at presentation, defined by a Mini‐Mental State Examination score <24, fulfilling DSM‐IV criteria for dementia 7 or Movement Disorder Society criteria for Parkinson’s disease dementia 8.

The study was approved by the Newcastle and North Tyneside Research Ethics Committee and performed according to the Declaration of Helsinki. All participants provided informed written consent.

### Assessments

Participants with PD were rated for disease severity by Hoehn & Yahr staging and motor severity using the Movement Disorder Society Unified Parkinson's Disease Rating Scale part III. Motor subtypes were defined as TD, PIGD and indeterminate type according to the methods described by Stebbins *et al*. 9. Due to the small numbers with indeterminate motor phenotype in the cohort, only comparisons between motor subtype of TD and PIGD were conducted. Levodopa equivalent daily dose (LEDD) was calculated for all dopaminergic medications using methods described by Tomlinson *et al*. 10.

Global cognition was assessed using the Mini‐Mental State Examination and Montreal Cognitive Assessment. Depressive symptoms were also assessed using the Geriatric Depression Scale‐15.

### Assessment of non‐motor symptoms and prodromal non‐motor symptoms

Participant NMS burden was assessed at the initial patient screening visit using the Non‐Motor Symptom Questionnaire (NMSQuest) 11. The NMSQuest is a 30‐item questionnaire that comprises 10 domains of NMS: gastrointestinal (GI) symptoms, urinary tract symptoms, sexual function, cardiovascular issues, depression/anxiety, sleep problems/fatigue, pain and a number of other complaints such as weight loss. A positive response about the presence of an NMS on the screening questionnaire elicited a further question about the estimated duration of the NMS symptom.

In order to determine the presence or absence of pNMS, we subtracted the duration of the presence of each individual NMS reported from the duration of earliest motor symptom. NMS whose duration preceded the duration of motor symptoms were considered a pNMS, whereas NMS that occurred after motor symptom onset were not classified as pNMS. The presence and duration of each NMS in excess of the duration of motor symptoms were recorded as a pNMS in months.

Individual pNMS symptoms were further classified into seven distinct non‐motor subtypes based on the symptom domains covered in the NMSQuest. These were GI symptoms, urinary tract symptoms, sexual dysfunction, cardiovascular symptoms, neuropsychiatric and cognitive symptoms, sleep dysfunction symptoms and miscellaneous symptoms. In order to be included in a prodromal non‐motor subtype, a participant had to have at least one pNMS consistent with that NMS domain.

### Statistical analysis

Statistical analyses were performed using SPSS software (Version 22, IBM Corp., Armonk, NY, USA). Data were assessed for normality using Kolmogorov–Smirnov tests. The mean and SD were computed for parametric variables, and the median and interquartile range for non‐parametric variables. Continuous and count data were compared using the parametric (*t*‐test) or non‐parametric (Mann–Whitney *U*‐test) test as appropriate and categorical data were compared with chi‐squared tests.

Hierarchical logistic regression was used to determine significant predictors of PIGD motor phenotype. Backwards stepwise logistic regression was used to produce a basic model of predictors involving age, gender and LEDD. Non‐significant predictors were excluded. Significant predictors were then included to give a basic model and total pNMS and pNMS domains (present or absent) were then individually added to the model. *P* < 0.05 was deemed to be significant for all analyses.

## Results

A total of 154 participants with a diagnosis of idiopathic PD were identified from the ICICLE‐PD cohort (Table 1). Participants had a mean age of 66.4 ± 10.4 years and had a median PD duration of 4.7 months [64.9% (*n* = 100) were male}.

**Table 1:** Characteristics of patients with Parkinson's disease (PD) (*n* = 154)

Variable	
Gender (male/female)	100 (64.9)/54 (35.1)
Age (years)	66.4 (10.4)
Duration of PD (months)	4.7 (2.6–8.1)
MDS‐UPDRS III score	26.9 ± 12.1
H&Y staging	2.0 ± 0.7
H&Y stage	
I	35 (22.7)
II	88 (57.1)
III	30 (19.5)
IV	1 (0.6)
V	0
PD medication treatment	
Drug naive	19 (12.3)
Levodopa	45 (29.2)
Dopaminergic agonists	57 (37)
MAOB inhibitor	73 (47.4)
LEDD (mg/day)	178.1 ± 148.2
Motor phenotype	
PIGD	78 (50.6)
Indeterminate	13 (8.4)
TD	63 (40.9)
MMSE score	28.6 ± 1.3
MoCA score^a^	25.2 ± 3.7
GDS‐15 score	2.8 ± 2.6

GDS‐15, Geriatric Depression Scale‐15; H&Y, Hoehn & Yahr; LEDD, levodopa equivalent daily dose; MAOB, monoamine oxidase B; MDS‐UPDRS, Movement Disorder Society Unified Parkinson's Disease Rating Scale, revised; MMSE, Mini‐Mental State Examination; MoCA, Montreal Cognitive Assessment; PIGD, postural instability gait difficulty motor subtype; TD, tremor dominant motor subtype. Data are given as mean ± SD, *n* (%) and median (interquartile range). ^a^MoCA completed in 140 patients with PD.

The presence or absence of pNMS was calculated [139 (90.3%) participants experienced pNMS] (Table 2). The median number of pNMS experienced by the patients with PD was 4 (interquartile range, 2–7). The most common individual pNMS experienced were as follows: hyposmia (39.6%), forgetfulness/memory complaints (36%), sialorrhea (33.8%), urinary urgency (30.2%) and anxiety (30.2%) (Table 2). Gender differences in pNMS experienced were observed, with males reporting significantly more sexual dysfunction (22% vs. 3.7%, respectively, *P* = 0.003), forgetfulness (38% vs. 22.2%, respectively, *P* = 0.046) and dream re‐enactment (29% vs. 14.8%, respectively, *P* = 0.049) compared with female participants (Table 2). Conversely, females reported significantly greater unexplained weight change (13% vs. 4%, respectively, *P* = 0.039) and anxiety (37% vs. 22%, respectively, *P* = 0.046).

**Table 2:** Prevalence of non‐motor symptoms (NMS) at screening and prodromal NMS (pNMS) in patients with Parkinson's disease (PD) and median duration before the development of motor symptoms

	pNMS (*n* = 154)	Time interval of NMS preceding motor symptoms (months)	pNMS in males vs. females			
			Males (*n* = 100)	Females (*n* = 54)	Test statistic	*P*‐value
No. of patients with PD with symptoms	139 (90.3)					
No pNMS experienced	4 (2–7)		4 (2–6.25)	4 (2–7)	*U* = 2850.5	0.649
Gastrointestinal tract						
Sialorrhea	47 (30.5)	−33 (−18 to −3)	34 (34)	13 (24.1)	*χ* ^2^ = 1.6	0.202
Dysphagia	18 (11.7)	−25 (−27 to −4)	11 (11)	7 (13)	*χ* ^2^ = 0.1	0.718
Nausea	3 (1.9)	−25 (−48 to −24)	1 (1)	2 (3.7)	*χ* ^2^ = 1.3	0.247
Constipation	38 (24.7)	−108 (−105 to −11)	24 (24)	14 (25.9)	*χ* ^2^ = 0.1	0.791
Bowel incontinence	4 (2.6)	−175 (−516 to −1)	2 (5)	2 (3.7)	*χ* ^2^ = 0.4	0.526
Incomplete bowel emptying	26 (16.9)	−55 (−31 to −6)	17 (17	9 (16.7)	*χ* ^2^ = <0.1	0.958
Hyposmia	55 (35.7)	−148 (−218 to −26)	32 (32.0)	23 (42.6)	*χ* ^2^ = 1.7	0.191
Weight change (unexplained)	11 (7.1)	−13 (−12 to −1)	4 (4.0)	7 (13.0)	*χ* ^2^ = 4.2	0.039
Urinary tract						
Urinary urgency	42 (27.3)	−63 (−65 to −12)	25 (25)	17 (31.5)	*χ* ^2^ = 0.7	0.389
Nocturia	29 (18.8)	−82 (−78 to −12)	18 (18)	11 (20.4)	*χ* ^2^ = 0.1	0.72
Sexual function						
Sexual dysfunction	24 (15.6)	−89 (−87 to −22)	22 (22.0)	2 (3.7)	*χ* ^2^ = 8.9	0.003
Impaired libido	15 (9.7)	−56 (−96 to −18)	12 (12)	3 (5.6)	*χ* ^2^ = 1.7	0.198
Cardiovascular						
Orthostatic symptoms	28 (18.2)	−23 (−31 to −2)	20 (20.0)	8 (14.8)	*χ* ^2^ = 0.6	0.426
Falls	16 (10.4)	−13 (−12 to −2)	11 (11.0)	5 (9.3)	*χ* ^2^ = 0.1	0.735
Lower limb swelling	17 (11.0)	−61 (−75 to −8)	10 (10.0)	7 (13.0)	*χ* ^2^ = 0.3	0.576
Neuropsychiatric and cognitive						
Forgetfulness/memory	50 (32.5)	−36 (−36 to −8)	38 (38)	12 (22.2)	*χ* ^2^ = 4.0	0.046
Impaired concentration	25 (16.2)	−37 (−33 to −8)	16 (16.0)	9 (16.7)	*χ* ^2^ = <0.11	0.915
Anxiety	42 (27.3)	−99 (−36 to −12)	22 (22.0)	20 (37.0)	*χ* ^2^ = 4.0	0.046
Low mood	27 (17.5)	−20 (−31 to −7)	15 (15)	12 (22.2)	*χ* ^2^ = <0.1	0.261
Loss of interest/apathy	22 (14.3)	−20 (−25 to −8)	13 (13)	9 (16.7)	*χ* ^2^ = 0.4	0.535
Delusions	0 (0)	NA	0 (0) NA	0 (0)	NA	NA
Visual hallucinations	12 (7.8)	−12 (−17 to −4)	7 (7.0)	5 (9.3)	*χ* ^2^ = 0.2	0.618
Sleep						
Daytime somnolence	32 (20.8)	−68 (−79 to −12)	25 (25)	7 (13)	*χ* ^2^ = 3.1	0.079
Insomnia	10 (6.5)	−28 (−51 to −11)	5 (5)	5 (9.3)	*χ* ^2^ = 1.0	0.306
Dream re‐enactment	37 (24.0)	−167 (−178 to −16)	29 (29.0)	8 (14.8)	*χ* ^2^ = 3.9	0.049
Vivid dream imagery	35 (22.7)	−175 (−228 to −12)	25 (25.0)	10 (18.5)	*χ* ^2^ = 0.8	0.36
Restless legs	28 (18.2)	−70 (−49 to −6)	14 (14.0)	14 (25.9)	*χ* ^2^ = 3.4	0.067
Miscellaneous						
Diplopia	11 (7.1)	−112 (−109 to−19)	6 (6.0)	5 (9.3)	*χ* ^2^ = 0.6	0.454
Hyperhydrosis	4 (2.6)	−48 (−93 to−11)	1 (1.0)	3 (5.6)	*χ* ^2^ = 2.9	0.090
Pain (unexplained)	32 (20.8)	−15 (−23 to−6)	17 (17)	15 (27.8)	*χ* ^2^ = 2.4	0.116

NA, not applicable. Data are given as *n* (%) and median (interquartile range).

Individual pNMS symptoms were classified into pNMS domains. The most frequent pNMS domains were GI tract (67.5%), sleep (52.6%), urinary tract (42.2%), cardiovascular system (32.5%) and miscellaneous systems (22.1%) (Table 3). In terms of the median duration of pNMS preceding motor symptom onset, sleep dysfunction (−66 months), sexual dysfunction (−60 months) and GI tract symptoms (−59  months) had the longest latency period (Table 3). When pNMS were grouped into symptom clusters, males experienced significantly more symptoms related to sexual problems compared with female participants (26% vs. 5.6%, respectively, *P* = 0.002, Table 3). Females experienced significantly more miscellaneous symptoms compared with male participants (40.7% vs. 19%, respectively, *P* = 0.004).

**Table 3:** Prevalence and median duration of prodromal non‐motor symptoms (pNMS) according to distinct pNMS domains

Symptom group	PD pNMS	Time interval of NMS preceding motor symptoms (months)	pNMS in males vs. females			
			Males (*n* = 100)	Females (*n* = 54)	*χ* ^2^	*P*‐value
Gastrointestinal tract	104 (67.5)	−58.5 (−217 to −16)	67 (67.0)	37 (68.5)	<0.1	0.848
Urinary tract	65 (42.2)	−45 (−126 to −14)	38 (38.0)	20 (37.0)	<0.1	0.906
Sexual function	32 (20.8)	−60 (−119 to −29)	26 (26.0)	3 (5.6)	9.6	0.002
Cardiovascular	50 (32.5)	−12 (−48 to −5)	35 (35.0)	15 (27.8)	0.8	0.361
Neuropsychiatric and cognitive	19 (12.3)	−39 (−132 to−14)	55 (55)	30 (55.6)	<0.1	0.947
Sleep	81 (52.6)	−66 (−237 to −19)	55 (55.0)	25 (46.3)	1.1	0.302
Miscellaneous	34 (22.1)	−12 (−48 to −8)	19 (19.0)	22 (40.7)	8.5	0.004

NMS, non‐motor symptoms; PD, Parkinson's disease. Data are given as *n* (%) and median (interquartile range).

Prodromal NMS differences between motor phenotypes were then evaluated (Table 4). Participants classified as having PIGD were significantly older than those with TD subtypes (68.4 ± 9.2 vs. 63.6 ± 11 years, respectively, *P* = <0.01; Table 4). The Geriatric Depression Scale‐15 scores were slightly higher for the PIGD subtype compared with the TD subtype (3.12 ± 2.4 vs. 2.27 ± 2.3, respectively, *P* = 0.01), but were still below the cut‐off (≥5) for possible depression in all subtypes. Participants with PIGD subtype were prescribed significantly higher doses of LEDD compared with TD subtype (203.0 ± 138.6 mg vs. 151.4 ± 165.4 mg, respectively, *P* < 0.01; Table 4).

**Table 4:** Demographics and prevalence of prodromal non‐motor symptoms among Parkinson's disease (PD) motor subtypes

Variable	PIGD (*n* = 78)	TD (*n* = 63)	Test statistic	*P*‐value
Gender (male/female)	56 (71.8)/22(28.2)	36 (57.1)/27 (42.9)	*χ* ^2^ = 3.3	0.069
Age (years)	68.4 (9.2)	63.6 (10.97)	*t* = 2.7	**0.007**
Duration of PD (months)	6.2 (4.8)	5.7 (4.4)	*U* = 2376.0	0.737
LEDD (mg/day)	203.04 ± 138.61	151.4 ± 165.4	*U* = 1684.5	**0.001**
MMSE score	28.53 (1.3)	28.8 (1.3)	*U* = 2081.0	0.106
MoCA score^a^	24.77 (3.6)	25.6 (3.7)	*U* = 1709.0	0.117
GDS‐15 score	3.12 (2.4)	2.3 (2.3)	*U* = 1849.5	**0.011**
MDS‐UPDRS III score	26.96 (12.1)	26.2 (12.1)	*U* = 2315.0	0.556

GDS‐15, Geriatric Depression Scale‐15; LEDD, levodopa equivalent daily dosage; MDS‐UPDRS, Movement Disorder Society Unified Parkinson's Disease Rating Scale, revised; MMSE, Mini‐Mental State Examination; MoCA, Montreal Cognitive Assessment; PIGD, postural instability gait difficulty motor subtype; TD, tremor dominant motor subtype. Data are given as mean ± SD and *n* (%) unless otherwise stated. Significant results are highlighted in bold. ^a^MoCA completed in 140 patients with PD.

Predictors of PIGD motor phenotype were then determined, first using logistic univariate regression. Significant predictors included age, LEDD, Geriatric Depression Scale‐15 score, number of pNMS, GI symptoms and urinary symptoms (Table S1).

Backwards regression revealed that only age [*β* = 0.061; odds ratio (OR), 1.06; 95% confidence interval (CI), 1.02–1.11, *P* = 0.003] was a significant predictor of PIGD phenotype; a basic model was then constructed with age and LEDD. LEDD was included in the basic model as it was an important confounding variable although it was not a significant predictor of PIGD phenotype. Total pNMS and pNMS domains were then individually added to the model. Analysis revealed that total pNMS was not a significant predictor of having PIGD phenotype (OR, 1.10; 95% CI, 0.99–1.21 *P* > 0.05). However, there was a significant association between any prodromal GI symptoms (OR, 2.30; 95% CI, 1.08–4.89, *P* < 0.05) and urinary symptoms (OR, 2.54; 95% CI, 1.19–5.35, *P* < 0.05) and the PIGD phenotype. Patients with PD with prodromal GI symptoms were thus 2.3 times more likely to develop PIGD phenotype, after controlling for age and LEDD. Similarly, participants with PD with prodromal urinary symptoms were 2.5 times more likely to evolve into a PIGD rather than TD phenotype, after controlling for age and LEDD (Supporting Information).

## Discussion

Our study strengthens the notion of NMS antedating motor symptoms in PD by assessing the presence and time of onset of the full spectrum of pNMS using a validated NMS questionnaire (NMSQuest) in a population with recently diagnosed PD. Interpretation of previous studies evaluating the frequency and duration of onset of pNMS is limited by study methodological variability, lack of direct patient evaluation 2, long duration from PD diagnosis to study enrolment 12, utilization of non‐validated, custom‐made NMS questionnaires 13 or studying only limited subsets of the spectrum on pNMS in PD cohorts 14 or ‘at risk’ of PD cohorts 15, 16.

Our study found 90% of participants reporting at least one pNMS, whereas the median number of pNMS experienced was four. Previous studies have reported higher prevalence of prodromal symptoms than our study, probably due to methodological differences. A retrospective study conducted via telephone interview with patients with PD and controls, using a custom‐made questionnaire, reported 98.9% of subjects with PD having one or more prodromal symptoms but this study incorporated prodromal motor symptoms as well as pNMS 2. Similarly, another retrospective study, with a long mean duration from PD diagnosis of 7.6 ± 5.6 years, reported 98.9% of subjects experienced prodromal symptoms preceding a diagnosis of PD 12.

Based on the Braak model of the hypothesized spread of alpha‐synuclein in PD, alpha synuclein accumulation begins in the gut before progressing via the vagus nerve to the brain 17. Therefore, GI features should be a prominent early manifestation of PD. Our study encompassed questions focusing on the GI tract that had not been previously reported, such as the prevalence of prodromal weight loss (7.1%), dysphagia (11.7%) and incomplete bowel emptying (16.9%). Prevalence of prodromal constipation symptoms (24.7%) and hyposmia (35.7%) have previously been reported and are approximately in line with published work 2, 13. Clustering prodromal GI symptoms together revealed that 67.5% of subjects with PD had one or more GI symptoms antedating motor symptom development in PD, which is consistent with the Braak model of early GI involvement in PD. Moreover, GI clusters, as well as urinary tract clusters, of pNMS were significantly associated with PIGD motor phenotype. To the best of our knowledge, no other study has shown an association between pNMS clusters and early PD motor phenotype.

In contrast with other studies, the prevalence of prodromal memory complaints (32.8%) and unexplained pain (20.8%) in our study is substantially higher compared with previous reported work 12, 13. Meanwhile, the prevalence of apathy (14.8%) and hyperhidrosis (2.6%) is significant lower than in other studies. The variability in reported prevalence rate of pNMS may be due to individual's perception and reporting of NMS. Many NMS progress slowly and are of mild severity, so are underappreciated in the early stages 12. Furthermore, cognitive impairment has also been associated with underestimation of NMS and loss of awareness of hyposmia has been reported to occur in Parkinson’s disease mild cognitive impairment 18. It is possible that cognitive performance may have impacted on symptom recall in our study as the mean Montreal Cognitive Assessment score was 25.2 (±3.7) and previously published work from the ICICLE‐PD study showed that, among the five cognitive domains, memory impairment was the most common domain affected in participants with PD at 1.5 SDs below normative values (15.1%) 5. However, all participants enrolled in the study underwent rigorous assessment to exclude dementia.

Gender has been reported as an independent predictor of NMS reported in early PD studies. Females have been reported to experience more anxiety, pain, depression and sleep disturbance 19, 20, 21, whereas males have been reported to experience more apathy and sexual dysfunction 22, 23, 24. However, there is a paucity of work examining the influence of gender on NMS in the prodromal period. We found a gender difference in terms of specific pNMS experienced, namely sexual dysfunction, forgetfulness and dream re‐enactment being more prevalent in males, and unexplained weight change and anxiety being more prevalent in females. Gender differences in perceived pNMS prevalence may be reflective of what each gender interprets to be important rather than any underlying early pathological evolutionary difference between genders.

There are some limitations in our study. The retrospective design of this study may have introduced recall error and thereby affected the accuracy of the data. Attempts were made to minimize recall errors by having experienced movement disorder physicians conduct face‐to‐face interviews with patients with recently diagnosed PD, including a caregiver interview. Another limitation is the lack of a validated prodromal questionnaire to evaluate pNMS. NMSQuest was adapted to identify pNMS as is has been extensively used to investigate NMS in *de‐novo*, early PD. However, its sensitivity and specificity for evaluating prodromal symptoms in ‘at risk’ PD cohorts have not been established. Therefore, it is entirely possible that some of the symptoms reported in this study are not related to an evolving Lewy body disorder but other underlying medical conditions or non‐specific normal age‐related symptoms.

In conclusion, our study has shown that pNMS are prevalent, antedate motor symptoms in some cases by several years and distinct gender differences exist in the pNMS experienced. Furthermore, prodromal GI and urinary tract symptoms were associated with the PIGD motor phenotype.

## Disclosure of conflicts of interest

R.D., R.A.L., T.K.K., G.W.D., N.P. and D.J.B. declare no financial or other conflicts of interest. L.W. declares no financial interests, patents or any other disclosures connected to this work. A.J.Y. reports other from Teva‐Lundbeck, grants from Newcastle NIHR BRC and other from UCB outside the submitted work. D.J.B. reports grants and personal fees from General Electric Healthcare and personal fees from Biogen outside the submitted work.

## Supporting information


**Table S1.** Logistic regression of predictors of PIGD phenotype versus TD phenotype.
