# Supplementary material for: Prevalence and duration of non‐motor symptoms in prodromal Parkinson's disease
Source: Eur J Neurol. 2019 Mar 1;26(7):979–85. doi: 10.1111/ene.13919 (PMC6563450; doi:10.1111/ene.13919)
Supplement: Supplementary file 1 — Table S1. Logistic regression of predictors of PIGD phenotype versus TD phenotype. [file ENE-26-979-s001.docx]

Supplemental Table: Logistic regression of predictors of PIGD phenotype versus TD phenotype

|  | | β | SE | p-value | OR | 95% CI for OR | |
| --- | --- | --- | --- | --- | --- | --- | --- |
|  |  |  |  |  |  | Lower Bound | Upper Bound |
| Univariate Analysis | |  |  |  |  |  |  |
|  | Age | 0.047 | 0.018 | 0.007 | 1.05 | 1.01 | 1.09 |
|  | Gender (male) | 0.647 | 0.358 | 0.071 | 1.91 | 0.95 | 3.85 |
|  | MDS-UPDRS III | 0.006 | 0.014 | 0.694 | 1.01 | 0.98 | 1.03 |
|  | LEDD | 0.002 | 0.001 | 0.050 | 1.00 | 1.00 | 1.01 |
|  | MMSE | 0.153 | 0.134 | 0.255 | 0.86 | 0.66 | 1.12 |
|  | MOCA | 0.064 | 0.50 | 0.204 | 0.94 | 0.85 | 1.04 |
|  | GDS-15 | 0.161 | 0.078 | 0.040 | 1.18 | 1.01 | 1.37 |
|  | Number of pNMS | 0.105 | 0.047 | 0.026 | 1.11 | 1.01 | 1.22 |
|  | Gastrointestinal symptoms | 0.981 | 0.369 | 0.008 | 2.67 | 1.29 | 5.50 |
|  | Urinary Symptoms | 0.893 | 0.363 | 0.014 | 2.44 | 1.20 | 4.98 |
|  | Sexual Symptoms | 0.650 | 0.467 | 0.164 | 1.92 | 0.77 | 4.79 |
|  | Cardiovascular Symptoms | 0.728 | 0.383 | 0.057 | 2.07 | 0.98 | 4.39 |
|  | Neuropsychiatric and cognitive Symptoms | 0.458 | 0.342 | 0.18 | 1.58 | 0.81 | 3.09 |
|  | Sleep Symptoms | 0.651 | 0.343 | 0.058 | 1.92 | 0.98 | 3.76 |
|  | Miscellaneous Symptoms | 0.536 | 0.396 | 0.176 | 1.71 | 0.79 | 3.72 |
| Basic model‡ + number of pNMS | |  |  |  |  |  |  |
|  | Number of pNMS | 0.090 | 0.050 | 0.068 | 1.10 | 0.99 | 1.21 |
| Basic model‡ + Gastrointestinal symptoms | |  |  |  |  |  |  |
|  | Gastrointestinal symptoms | 0.834 | 0.384 | **0.030** | 2.30 | 1.08 | 4.89 |
| Basic model‡ + Urinary symptoms | |  |  |  |  |  |  |
|  | Urinary symptoms | 0.926 | 0.383 | **0.016** | 2.54 | 1.19 | 5.35 |
| Basic model‡ + Sexual symptoms | |  |  |  |  |  |  |
|  | Sexual symptoms | 0.471 | 0.487 | 0.333 | 1.60 | 0.62 | 4.16 |
| Basic model‡ + Cardiovascular symptoms | |  |  |  |  |  |  |
|  | Cardiovascular symptoms | 0.438 | 0.405 | 0.280 | 1.55 | 0.70 | 3.43 |
| Basic model‡ + Neuropsychiatric and cognitive | |  |  |  |  |  |  |
|  | Neuropsychiatric and cognitive | 0.358 | 0.356 | 0.315 | 1.43 | 0.71 | 2.88 |
| Basic model‡ + Sleep symptoms | |  |  |  |  |  |  |
|  | Sleep symptoms | 0.668 | 0.359 | 0.063 | 1.95 | 0.965 | 3.95 |
| Basic model‡ + Miscellaneous symptoms | |  |  |  |  |  |  |
|  | Miscellaneous symptoms | 0.548 | 0.418 | 0.190 | 1.73 | 0.76 | 3.93 |

‡ Basic model comprises age and levodopa equivalent dose

Significant p-values highlighted in bold

Abbreviations: PIGD = Postural instability gait difficulty motor subtype; TD = Tremor dominant motor subtype; NMS= Non-motor symptoms; pNMS= prodromal non-motor symptoms;
